# Adding pegylated interferon-α to nucleos(t)ide analogs improves HBsAg clearance in chronic hepatitis B with low-level viremia

**DOI:** 10.3389/fmed.2025.1642961

**Published:** 2025-08-22

**Authors:** Lilin Wang, Junfeng Lu, Dan Liu, Shan Ren, Xinyue Chen, Zhongjie Hu, Sujun Zheng

**Affiliations:** The First Department of Liver Disease Center, Beijing You’an Hospital, Capital Medical University, Beijing, China

**Keywords:** low-level viremia, chronic hepatitis B, pegylated interferon-α, tenofovir alafenamide fumarate, HBsAg clearance

## Abstract

**Introduction:**

Low-level viremia (LLV) is associated with the progression of liver fibrosis and a high risk of hepatocellular carcinoma in patients with chronic hepatitis B (CHB). The present study aimed to compare the efficacy between nucleos(t)ide analogs (NAs) therapy and combination therapy of NAs and pegylated interferon-α (pegIFN-α) in entecavir (ETV)-treated CHB patients with LLV.

**Methods:**

This was a retrospective cohort study. ETV-treated CHB patients with LLV were included and divided into the NA group and the NA+IFN group. The NA group comprised patients switching to tenofovir alafenamide fumarate, whereas the NA+IFN group comprised those adding on pegIFN-α additionally. We compared changes in HBV markers and complete virological response (CVR) between the two groups.

**Results:**

A total of 127 patients were enrolled, including 51 in NA+IFN group and 76 in NA group. In the NA+IFN group, the decline in HBsAg level from baseline (△ HBsAg) was significantly greater (−0.17 log_10_IU/mL vs. −0.06 log_10_IU/mL, *P* = 0.011) at week 24, and HBsAg clearance rate and △ HBsAg were significantly higher (8.9% vs. 0%, *P* = 0.017; −0.27 log_10_IU/mL vs. −0.11 log_10_IU/mL, *P* = 0.023) at week 48. The 48-week CVR rate in the NA+IFN group was 66.7% (34/51), which was comparable to 68.4% (52/76) in the NA group (*P* = 0.836).

**Conclusion:**

In ETV-treated patients with LLV, receiving NAs plus pegIFN-α tends to increase the effect of HBsAg clearance.

## 1 Introduction

Hepatitis B virus (HBV) plays an important role in the pathogenesis and progression of chronic hepatitis B (CHB), fibrosis, cirrhosis, and hepatocellular carcinoma (HCC). Approximately 257 million people are chronically infected with HBV, and 8.87 million patients die from HBV-related cirrhosis, liver failure, and HCC each year worldwide (1). Antiviral therapy improves the clinical outcome and reduces the incidence of HCC among patients with CHB (2–4). Therefore, effectively suppressing the replication of HBV is crucial.

Despite high-genetic-barrier antiviral agents, some patients retain low HBV DNA levels. Complete virological response (CVR) is defined as persistently undetectable HBV DNA levels on therapy. Both US and European guidelines indicate that even with first-line therapies, treatment-naïve HBeAg-positive CHB patients achieve CVR rates around 70% (3, 4). The American Association for the Study of Liver Diseases (AASLD) has defined low-level viremia (LLV) as HBV DNA < 2,000 IU/mL that is nevertheless detectable during therapy (4). Over the years, virological tools for measuring HBV DNA have improved, and thresholds for an undetectable HBV DNA level have decreased. Thus, patients with sustained virological response as examined using assays of the past may actually present with LLV when using high-sensitive HBV DNA assays. Evidence from a cross-sectional study indicates that incomplete virological suppression is associated with abnormal liver histopathology (5). Another study has suggested that LLV may interfere with fibrosis regression and even promote fibrosis progression in CHB (6). Previous research has indicated that LLV is an independent risk factor of HCC (7–10). Moreover, the tumor recurrence and mortality rates of HCC are relatively high in patients with LLV (11).

Entecavir (ETV) is recommended as one of the first-line drugs based on its high potency and low frequency of developing resistance (3, 4, 12). Several real-world studies have shown that 20%–40% of long-term ETV-treated patients remain in a state of LLV (13, 14). Although the guidance recently updated by the AASLD suggests that patients with LLV treated with ETV or tenofovir monotherapy should continue monotherapy, the quality and certainty of the evidence are very low (4). It remains to be determined how ETV-treated patients with LLV should be managed. In patients with persistent LLV, after correcting for other factors such as poor adherence and alcohol intake, optimizing therapeutic options is necessary for improving the CVR rate and long-term prognosis (6). Zhang et al. (15) demonstrated that patients switching therapy achieved higher CVR rates than those continuing original regimens, with superior long-term outcomes (15). Therefore, patients with LLV need to switch to a more effective antiviral therapy in time. Nevertheless, hitherto there is no consensus about the effective therapeutic strategies for this kind of patients.

Compared with the development of novel HBV inhibitors, utilizing an available treatment is cost-effective and potentially a more viable option. Current therapeutic options are still limited to nucleos(t)ide analogs (NAs) and pegylated interferon-α (pegIFN-α).

In recent years, tenofovir alafenamide fumarate (TAF) has been listed as the first-line anti-HBV drug in the guidelines for the prevention and treatment of CHB in China, USA, and Europe (12, 16). However, NA therapy is unable to eliminate covalently closed circular DNA in hepatocytes and has difficulty achieving HBsAg clearance. In addition, drug resistance may occur after a long course of NA therapy. Therefore, switching to or adding on another NA drug is not an effective therapeutic regimen for good and all. PegIFN-α is a combination of interferon-α and polyethylene glycol, which has a more persistent and potent antiviral effect. Moreover, interferon-α can inhibit cell proliferation, thereby playing a role in preventing the development of HCC in CHB patients (17). PegIFN-α therapy frees the patients from the predicament of multidrug resistance. Data on the efficacy and safety of combination therapy of NAs and pegIFN-α in this type of patient are scarce. Limited data exist on pegIFN-α efficacy in LLV patients. We retrospectively analyzed ETV-treated CHB patients with LLV to assess combination therapy of NA and pegIFN-α.

## 2 Materials and methods

### 2.1 Study population

This was a retrospective cohort study. ETV-treated CHB patients with LLV between December 2018 and August 2022 in Beijing You’an Hospital, Capital Medical University, were eligible. The inclusion criteria were as follows: (1) age > 18 years; (2) hepatitis B surface antigen (HBsAg) positivity for more than 6 months or by clinical history; (3) ETV treatment received for more than 48 weeks; and (4) persistently low levels (< 2,000 IU/mL, but detectable) of HBV DNA in at least two consecutive assessments at every 12-week interval by high-sensitive PCR in the year before the enrolment. The exclusion criteria were as follows: (1) history or current signs of liver cirrhosis and HCC; (2) concomitant other liver diseases (coinfection with hepatitis A virus, hepatitis C virus, hepatitis D virus, hepatitis E virus, cytomegalovirus or Epstein–Barr virus, alcoholic fatty liver disease, non-alcoholic fatty liver disease, and autoimmune hepatitis); (3) coinfection with human immunodeficiency virus; (4) peripheral white blood cell count < 3.0 × 10^9^/L and/or neutrophil count < 1.0 × 10^9^/L and/or platelet count < 75 × 10^9^/L, total bilirubin > 34 μmol/L, or creatinine > 1.5× upper limit of normal; (5) diagnosis of thyroid disease, autoimmune disease, other serious organic diseases or malignancy, severe neurological or psychological disease; and (6) pregnancy or lactation in women.

The enrolled patients were divided into the NA group and the NA+IFN group based on the patients’ own discretion. The NA group comprised patients switching to tenofovir alafenamide fumarate (TAF), whereas the NA+IFN group comprised those adding on pegIFN-α additionally.

The study was performed in accordance with The Code of Ethics of the World Medical Association (Declaration of Helsinki) and was approved by the Ethics Committee of Beijing You’an Hospital, Capital Medical University (code: LL-2020-174-K).

### 2.2 Measurements

Baseline demographic data were obtained. Laboratory assessments encompassing virological/biochemical parameters were performed at baseline, week 24 and week 48. HBV DNA quantification employed the Roche Cobas/Taqman qPCR system (Germany; LLOD 10 IU/mL). CVR was defined as serum HBV DNA < 10 IU/mL. Serological markers were assessed via Roche E601 chemiluminescence analyzer (HBsAg LLOD 0.05 IU/mL; HBeAg LLOD 1 COI).

At week 48, we compared CVR rates, HBeAg/HBsAg clearance rates, and both baseline levels and changes in serum HBV DNA, HBsAg and HBeAg between the two groups.

### 2.3 Statistical analysis

For categorical variables, data were presented as frequencies and percentages. The normality of continuous variables was assessed using the Shapiro–Wilk test. Normally distributed continuous variables were expressed as means with standard deviations (SDs), while non-normally distributed ones were reported as medians with interquartile ranges (IQRs). Comparisons of categorical variables were carried out using the chi-square test or Fisher’s exact test. The Mann–Whitney U test was used for continuous variables that were not normally distributed, while the independent-samples *t*-test was used for continuous variables that followed a normal distribution.

All statistical tests were two-tailed, and *P*-values lower than 0.05 were considered statistically significant. Statistical analyses were conducted using SPSS 25.0.

## 3 Results

### 3.1 Baseline characteristics

A total of 127 ETV-treated CHB patients with LLV were enrolled in this study, including 82 men and 45 women. Of the 127 patients, 76 (59.8%) in the NA group and 51 (40.2%) in the NA plus pegIFN-α combination group. Baseline characteristics were comparable between the two groups (all *P* > 0.05), except for age (Table 1).

**TABLE 1:** Baseline characteristics of the study population.

Variable	Overall (*n* = 127)	NA+IFN (*n* = 51)	NA (*n* = 76)	*P*
Age, years	41.0 (34.0, 49.5)	36.0 (32.5, 44.0)	44.5 (36.8, 55.3)	< 0.001*
Female sex, *n* (%)	45 (38.5)	17 (33.3)	28 (36.8)	0.709
HBV DNA, log_10_IU/mL	1.77 (1.34, 2.23)	1.80 (1.46, 2.16)	1.75 (1.32, 2.33)	0.807
HBsAg, log_10_IU/mL	3.42 (2.98, 3.81)	3.42 (3.01, 3.75)	3.40 (2.97, 3.84)	0.854
HBeAg, log_10_COI	0.55 (−0.95, 2.18)	0.16 (−0.98, 1.72)	0.95 (−0.31, 2.34)	0.086
HBeAg positive *n* (%)	74 (63.2)	28 (56.0)	46 (66.7)	0.584
ALT, U/L	23.0 (14.3, 31.0)	26.0 (16.0, 30.0)	22.0 (14.0, 32.0)	0.748
AST, U/L	24.5 (21.0, 31.0)	25.0 (21.0, 30.5)	24.0 (20.0, 31.5)	0.758
TBIL, μmol/L	15.7 (13.1, 19.9)	15.6 (12.4, 19.4)	15.7 (13.6, 20.1)	0.678
PLT, ×10^9^/L	215.0 ± 57.6	204.2 ± 57.8	223.5 ± 56.5	0.077

ALT, alanine aminotransferase; AST, aspartate aminotransferase; TBIL, total bilirubin; PLT, platelet count. *P < 0.05.

### 3.2 Antiviral efficacy

#### 3.2.1 Virological response

**FIGURE 1:**
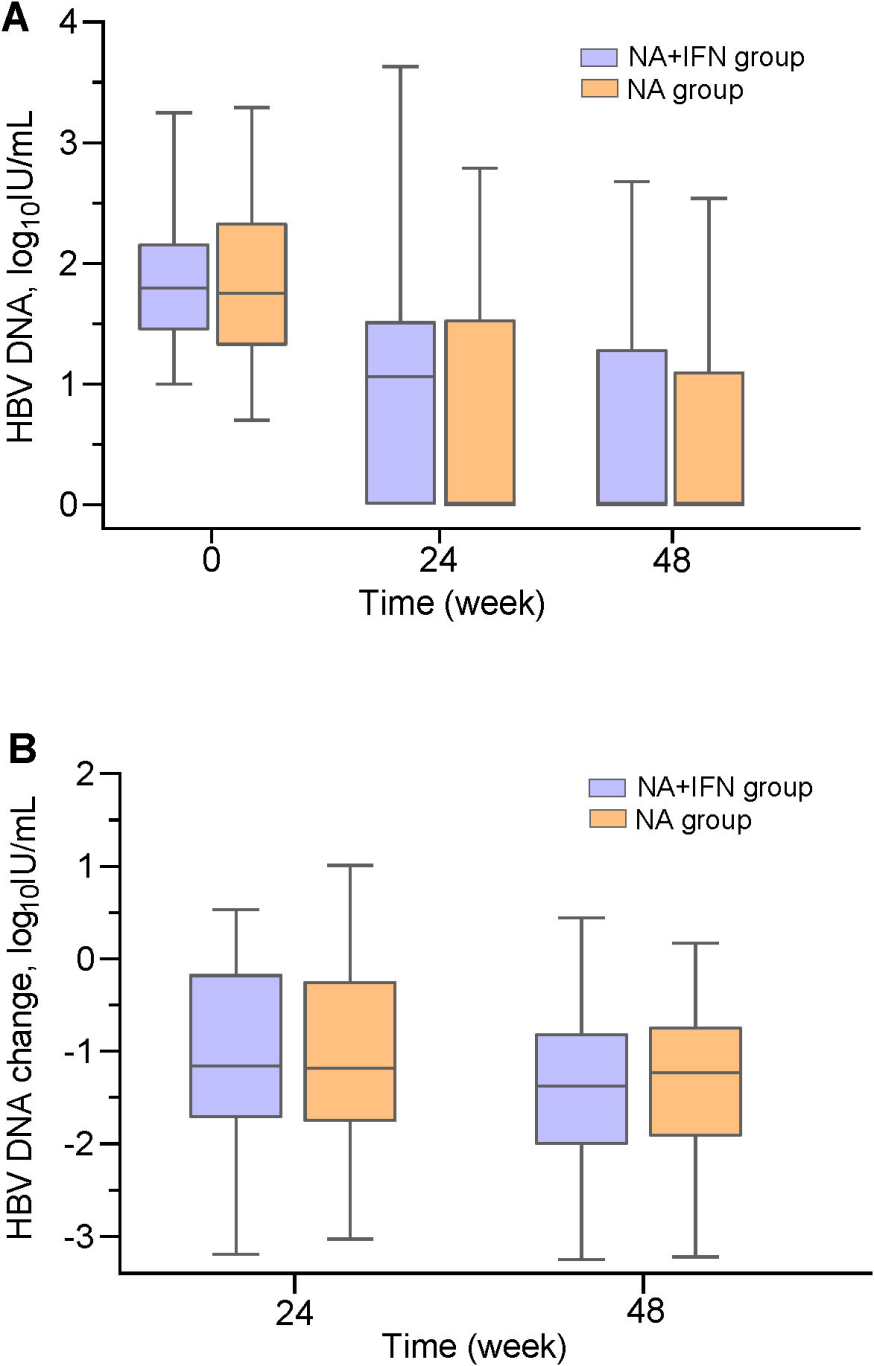
HBV DNA dynamics during follow-up. **(A)** Serial HBV DNA levels in both groups throughout the study period. **(B)** HBV DNA change from baseline at weeks 24 and 48.

Baseline HBV DNA levels were comparable between the two groups (*P* = 0.807). During the 48-week therapy, HBV DNA levels decreased over time in both groups (*P* < 0.05) (Figure 1A). However, by week 48, HBV DNA levels in the NA group were comparable to those in the NA+IFN group (*P* = 0.874). The reduction in HBV DNA levels from baseline showed no significant difference between the two groups at both week 24 and 48 (Table 2 and Figure 1B).

**TABLE 2:** Efficacy evaluation.

	Week 24			Week 48		
Variable	NA+IFN (*n* = 51)	NA (*n* = 76)	*P*	NA+IFN (*n* = 51)	NA (*n* = 76)	*P*
CVR, *n* (%)	25 (49.0)	43 (56.6)	0.402	34 (66.7)	52 (68.4)	0.836
HBV DNA, log_10_IU/mL	1.06 (0, 1.52)	0 (0, 1.53)	0.516	0 (0, 1.28)	0 (0, 1.1)	0.874
HBV DNA change, log_10_IU/mL	−1.15 (−1.71, 0.18)	−1.18 (−1.75, 0.25)	0.530	−1.38 (−2, 0.83)	−1.23 (−1.89, 0.74)	0.861
HBsAg clearance, *n*/*N* (%)	0	0	NA	4/45 (8.9)	0/62 (0)	0.017
HBsAg, log_10_IU/mL	3.33 (2.61, 3.74)	3.32 (2.91, 3.65)	0.625	3.2 (2.28, 3.73)	3.17 (2.82, 3.61)	0.661
HBsAg change, log_10_IU/mL	−0.17 (−0.47, 0.05)	−0.06 (−0.17, 0)	0.011*	−0.27 (−0.66, 0.05)	−0.11 (−0.22, 0.04)	0.023*
HBeAg clearance, *n*/*N* (%)^a^	1/27 (3.7)	3/35 (8.6)	0.139	4/24 (16.7)	5/37 (13.5)	0.734
HBeAg, log_10_COI	0.12 (−1.05, 1.43)	−0.06 (−1.08, 1.24)	0.123	0.76 (−0.78, 1.92)	0.72 (−0.85, 1.75)	0.038*
HBeAg change, log_10_COI^a^	−0.12 (−0.27, −0.02)	−0.21 (−0.41, −0.05)	0.921	−0.11 (−0.23, −0.4)	−0.17 (−0.47, −0.07)	0.942

*^a^*Among patients who were seropositive for HBeAg and negative for anti-HBeAg at baseline. *P < 0.05.

The CVR rates increased after altering antiviral therapy in each group. The CVR rate in the NA group was comparable to that in the NA+IFN group at both week 24 and 48. Specifically, at week 24, the CVR rates were 56.6% (43/76) and 49.0% (25/51), respectively, (*P* = 0.402). By week 48, the CVR rates increased to 68.4% (52/76) and 66.7% (34/51), respectively, (*P* = 0.836) (Table 2).

#### 3.2.2 HBsAg clearance

**FIGURE 2:**
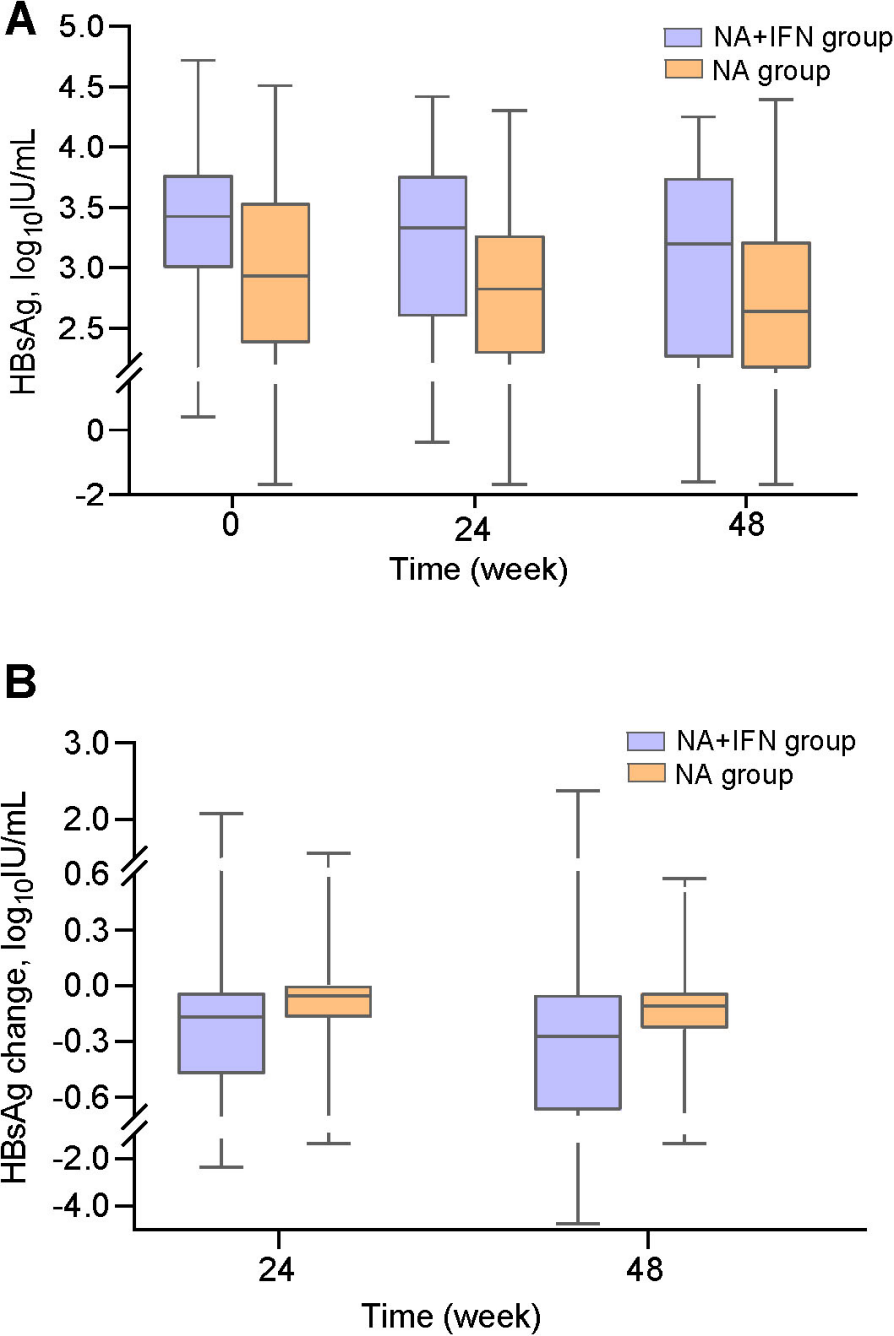
HBsAg dynamics during follow-up. **(A)** HBsAg levels in both groups throughout the study period. **(B)** HBsAg level change from baseline at weeks 24 and 48.

The two groups had comparable baseline HBsAg levels (*P* = 0.854, Table 1). Although 48-week HBsAg levels showed no intergroup difference, the NA+IFN group demonstrated significantly greater reductions from baseline at both week 24 (*P* = 0.011) and week 48 (*P* = 0.023) compared to the NA group (Table 2 and Figure 2). Furthermore, compared with the NA group, the 48-week HBsAg clearance rate was significantly higher in the NA+IFN group (8.9% vs. 0%, *P* = 0.017). In the NA group, none of the patients showed complete disappearance of serum HBsAg during the 48-week treatment period. Four patients achieved HBsAg clearance in the NA+IFN group at week 48.

#### 3.2.3 HBeAg clearance

**FIGURE 3:**
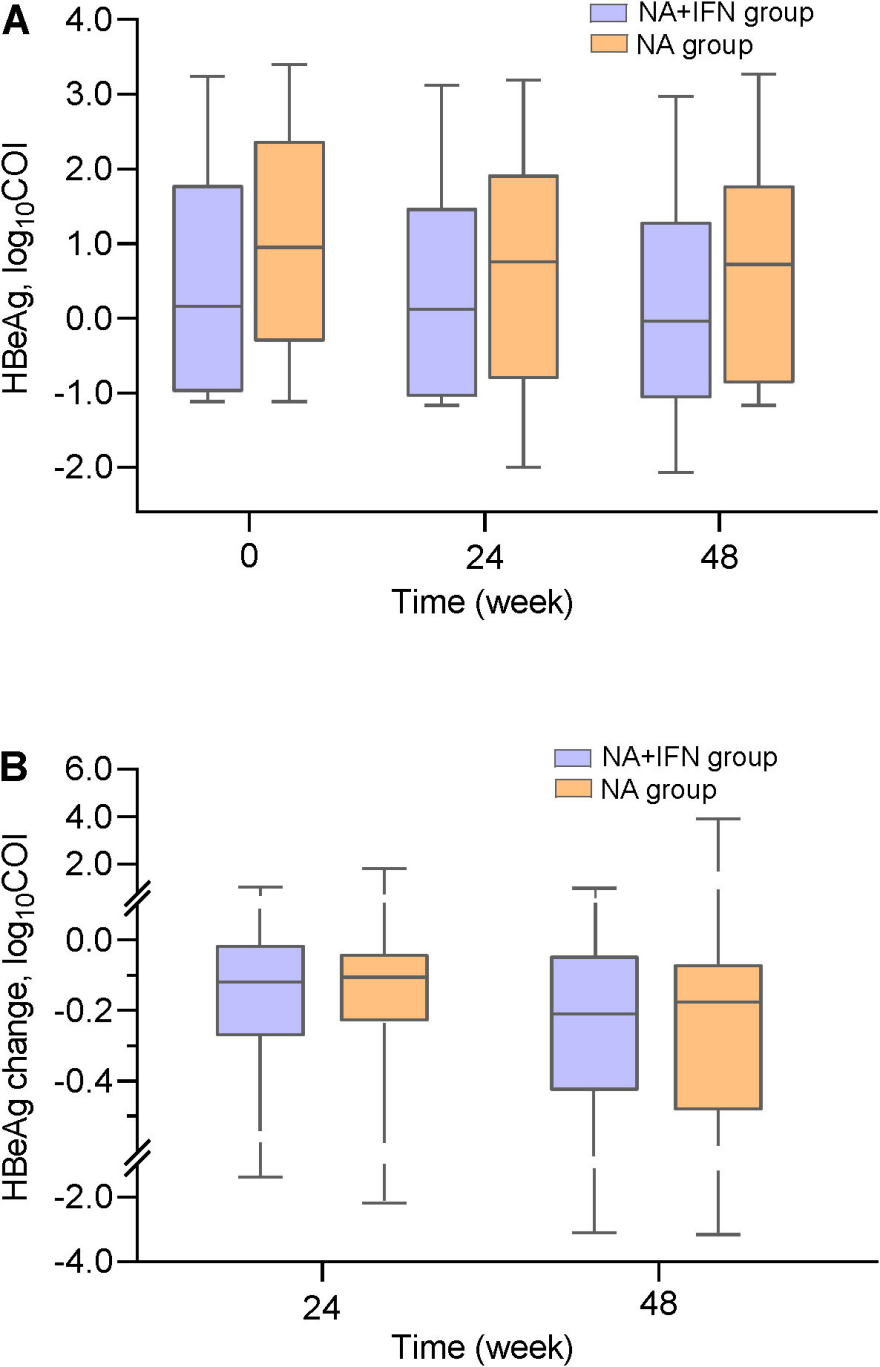
HBeAg dynamics during follow-up. **(A)** HBeAg levels in both groups throughout the study period. **(B)** HBeAg level change from baseline at weeks 24 and 48.

Baseline HBeAg levels were comparable between the two groups (*P* = 0.086, Table 1). A significant difference in HBeAg levels was observed between the two groups at week 48 (*P* = 0.038, Table 2 and Figure 3A). Compared with the NA group, the reduction in HBeAg was comparable to that of NA+IFN group at week 48 (*P* = 0.942, Table 2 and Figure 3B). The HBeAg clearance rates increased with time after altering antiviral therapy in both groups. The 48-week HBeAg clearance rate among HBeAg-positive patients in the NA group was 13.5% (5/37), which was not significantly different from the rate of 16.7% (4/24) in the NA+IFN group (*P* = 0.734) (Table 2).

### 3.3 Safety

During the follow-up period, both therapies were well tolerated. There was no drug discontinuation due to adverse events. We analyzed white blood cell count (WBC) and platelet count (PLT) among patients after adding on pegIFN-α. At week 24, leukopenia, thrombocytopenia, and alanine aminotransferase (ALT) elevation occurred in 68.6, 51, and 43.1% of the patients, respectively. However, the rates of leukopenia, thrombocytopenia and ALT elevation decreased at week 48 (Table 3).

**TABLE 3:** Incidence of adverse reactions in the NA+IFN group.

Adverse reactions	Week 24	Week 48
Leukopenia, n/N (%)	35/51 (68.6)	20/51 (39.2)
Thrombocytopenia, n/N (%)	26/51 (51.0)	18/51 (35.3)
ALT elevation, n/N (%)	22/51 (43.1)	6/51 (11.8)

ALT, alanine aminotransferase.

## 4 Discussion

In patients with chronic HBV infection, the major therapeutic targets of antiviral therapy are to suppress HBV replication, inhibit hepatitis activity, reverse cirrhosis, and improve liver function (18). LLV remains a noteworthy issue (13, 19–21) and sheds light on the current plight of NAs therapy. Therefore, we compared the anti-HBV efficacy between NAs and combination therapy with NAs and pegIFN-α. This retrospective cohort of ETV-treated CHB patients with LLV provided evidence of the effectiveness and advantage of combination therapy with NAs and pegIFN-α. The results showed that 48-week combination therapy with NAs and pegIFN-α improved clearance of HBsAg in ETV-treated patients with LLV. The HBsAg level also significantly declined among patients receiving NAs plus pegIFN-α. However, compared with NA therapy, adding on pegIFN-α for 48 weeks did not enhance the effect on suppressing virus.

TAF is a phosphonoamidate prodrug of tenofovir, a nucleotide reverse-transcriptase inhibitor. Previous studies have demonstrated the benefits of switching from ETV to TAF (22, 23). CVR is not only the precondition for biological remission, histological improvement, and preventing disease progression, but also the direct criterion for evaluating the effect of anti-HBV therapy (24). Recent studies have shown that switching to TAF significantly improves virological response in patients with LLV treated with ETV or NAs combination therapy (7, 14, 25–28). The evidence suggests that switching to TAF benefits prognosis and should be considered an alternative treatment strategy for ETV-treated patients with LLV. In this study, the 48-week CVR rate in the NA group was 68.4%, aligning with previous findings. Additionally, the decline in HBV DNA from baseline is consistent with a prospective study in China (7).

As pegIFN-α and NAs target different sites of HBV, they do not exhibit competitive inhibition. Clinical studies have confirmed that their combination therapy enhances clinical cure by synergistically inhibiting viral replication and modulating host immunity (29–31). Given the persistent immunoregulatory effect of pegIFN-α, we speculated that combining NAs with pegIFN-α could reduce HBV DNA, HBeAg, and HBsAg levels, thereby enhancing HBeAg and HBsAg clearance rates in patients with LLV.

We investigated CVR rates and the dynamic changes in HBV DNA levels from baseline to week 48. Our data showed a time-dependent increase in CVR rates in both groups. However, no significant differences were observed in CVR rates or the decline in HBV DNA between the two groups at any time point. These findings suggest that adding pegIFN-α may not enhance viral inhibition, possibly due to the poor immunity in patients with LLV. Immune response triggers liver inflammation and ALT elevation (1). Previous studies have indicated that pronounced liver inflammation facilitates better virological response after antiviral therapy (32, 33). In our study, baseline ALT levels were relatively low, potentially reflecting weak immune responses. In LLV patients, an insufficient host immune response fails to effectively eliminate infected hepatocytes. Consequently, even with pegIFN-α, no significant improvement in CVR rates was observed. This aligns with findings from another Chinese study (34), suggesting that the antiviral efficacy of switching to TAF is comparable to that of add-on TAF.

Loss of serum HBsAg and/or seroconversion indicates resolution of HBV infection and is considered the optimal endpoint of antiviral therapy, referred to as a “functional cure” (3). However, achieving HBsAg loss is challenging in the presence of integrated HBV DNA-driven expression of viral envelope proteins. In patients with LLV, reaching the goal of HBsAg clearance is difficult, even with 48 weeks of pegIFN-α combined with NAs. In our study, four patients in the NA+IFN group achieved HBsAg clearance, whereas no patients in the NA group did so at week 48. After 48 weeks of treatment, the NA+IFN group demonstrated a significantly higher HBsAg clearance rate compared to the NA group (8.9% vs. 0%, *P* = 0.017). These findings suggest that combination therapy with pegIFN-α and NAs may enhance HBsAg clearance in patients. We found that the decline in HBsAg level from baseline in the NA group was similar to a previous study (14). In our study, the NA+IFN group exhibited a significantly greater reduction in HBsAg levels compared to the NA group at both week 24 (−0.17 vs. −0.06 log_10_IU/mL, *P* = 0.011) and week 48 (−0.27 vs. −0.11 log_10_IU/mL, *P* = 0.023). These results show that 48 weeks of combination therapy with pegIFN-α and NAs had an advantage over NAs in reducing HBsAg levels, particularly in the early stages of treatment. This suggests that long-term combination therapy of NAs with pegIFN-α may benefit the functional cure of patients with LLV.

Our results align with previous studies, which have shown that, compared to NAs, pegIFN-α offers an advantage in clearing HBsAg without the risk of drug resistance. However, the inhibitory effect of pegIFN-α on HBV DNA replication is weaker than that of NAs (3, 35).

In terms of safety, no serious adverse events or treatment discontinuations due to adverse events were reported in our study. We analyzed common blood-related adverse reactions and found that neutropenia and thrombocytopenia occurred in more than half of the patients receiving combination therapy with pegIFN-α and NAs. However, the rates of neutropenia and thrombocytopenia at week 48 were lower than at week 24. The incidence of ALT elevation followed a similar trend. In other words, WBC, PLT, and ALT levels returned to normal in some patients over time, suggesting that pegIFN-α therapy is well tolerated in patients with LLV.

Our study has several limitations. First, it was a single-center study with a limited number of cases. Potential selection bias may exist due to the exclusion of patients lost to follow-up or with missing data, possibly affecting generalizability. Second, due to ethical considerations, we did not include a control group continuing ETV therapy. Previous studies have shown that, compared to patients changing treatment regimens, those continuing ETV therapy had relatively low virological and serological response rates. Third, genotype data were unavailable, though it is well known that the most common HBV genotypes in the Asia-Pacific region are B and C. Fourth, we did not investigate the development of drug-associated mutations. LLV may indicate saturation or competitive inhibition in the absence of drug resistance. Moreover, given the good patient compliance and the very low resistance rate of ETV, HBV mutations are unlikely to be a major factor in persistent LLV during antiviral therapy. Finally, histopathological data were not available.

## 5 Conclusion

In conclusion, this retrospective real-world cohort study demonstrated a trend toward increased clearance of HBsAg in ETV-treated patients with LLV receiving NAs plus pegIFN-α. However, combination therapy with NAs and pegIFN-α may not offer any advantage in virological response compared to NA therapy alone.

## Data Availability

The datasets presented in this article are not readily available because our dataset contains sensitive medical data of patients; hence data are not publicly available. Anonymized data that support the findings of this study are available from the corresponding author upon reasonable request. Requests to access the datasets should be directed to zhengsujun003@163.com.
